# Sleep disturbance among frontline nurses during the COVID-19 pandemic

**DOI:** 10.1007/s41105-021-00337-6

**Published:** 2021-07-01

**Authors:** Mohammed Al Maqbali

**Affiliations:** 1 Department of Nursing Midwifery and Health, Northumbria University, Newcastle-Upon-Tyne, UK; 2 Al Buraimi Hospital Ministry of Health, Al Buraimi, Oman

**Keywords:** COVID-19, Frontline nurses, Sleep disturbance, PSQI, Stress, Anxiety, Depression

## Abstract

The main objective of this study is to assess the prevalence of sleep disturbance and related psychological factors (stress, anxiety and depression) among frontline nurses in Oman during the COVID-19 pandemic. A cross-sectional and descriptive correlational design using Qualtrics® software was used in this research. Data were collected using the Pittsburgh Sleep Quality Index (PSQI), the Hospital Anxiety and Depression Scale (HADS) and the Perceived Stress Scale (PSS). Logistic regression was performed to identify factors associated with poor sleep quality. Of the 987 frontline nurses who participated, 58.8% (*n* = 580) reported poor sleep quality. In an examination of PSQI components the mean sleep duration was 7.04 (SD = 1.59) hours per night, and the sleep latency mean was 38.18 min (SD = 31.81). Poor sleep (*p* < .05) was significantly associated with age, marital status, years of experience, comorbidity, and whether family members or relatives were suspected or confirmed with having COVID-19. Logistic regression showed that poor quality of sleep was significantly associated with stress, anxiety and depression symptoms. Sleep disturbance is a significant problem for frontline nurses working in Oman during the COVID-19 pandemic. Appropriate interventions to maintain the health conditions and reduce sleep disturbance among frontline nurses are needed in order to help support nurses’ work during contagious disease outbreaks. These can be implemented through online workshops and training to enhance nurses’ responses to the pandemic or to any further disease outbreaks.

## Introduction

Severe acute respiratory syndrome coronavirus was identified at the end of December 2019 in Wuhan City, Hubei province, China, and has since spread worldwide [1]. On 11 February 2020, the World Health Organization (WHO) renamed the disease as the Corona Virus Disease-2019 (COVID-19) [2]. COVID-19 seriously threatens human health as it can be transmitted via close human-to-human contact. On 30th January 2020, a public health emergency was declared by the WHO, naming COVID-19 a pandemic [3]. As of 31st May 2021, a total of more than 170 million cases have been confirmed worldwide, with more than 3.5 million deaths [4].

This extreme number of patients can overwhelm healthcare systems with thousands of patients needing urgent care. This increases the workload on healthcare workers, especially frontline nurses who are in direct contact with patients with COVID-19. In June 2020, the International Council of Nurses (ICN) estimated that more than 600 nurses worldwide have died from COVID-19 [5]. Therefore, frontline nurses working in situations with a high risk of infection from patients can lead to sleep disturbance, stress, anxiety and depression.

Previous studies have reported the prevalence of sleep disturbance outcomes among healthcare workers during SARS and Middle East Respiratory Syndrome (MERS) outbreaks [6–8]. Healthcare workers, particularly nurses, are at high risk of being infected because they are in the closest proximity to patients. Current research has already shown that Chinese nurses are suffering from sleep disturbance due to the COVID-19 pandemic [9].

However, to date, little has been known about the impact of the COVID-19 outbreak on the incidence of sleep disturbance among frontline nurses. This study will help to clarify the sleep disturbance status of frontline nurses, knowledge of which is crucial to better controlling and planning for dealing with COVID-19 or similar diseases in the future. The aim of this study is to assess the prevalence of sleep disturbance and related psychological factors (stress, anxiety and depression) among frontline nurses in Oman during the COVID-19 pandemic.

## Methods

### Study design

The study employed a large-scale cross-sectional, descriptive correlational design. The survey was developed using the Qualtrics^®^ online platform. Participants were invited to complete the questionnaire through a link which was sent by social media.

### Setting and sampling

The participants were recruited from all Ministry of Health institutions in Oman. The study was performed from 7th August 2020 to 30th August 2020. The inclusion criteria for participating in the study were: being a nurse who worked in a Ministry of Health institution; being aged older than 18 years; and being frontline nurses (in contact with confirmed or suspected COVID-19 patients in the workplace). Exclusion criteria included a history of psychiatric or neurological disorders that could interfere with participation in the study, and nurses who do not have any working contact with COVID-19 patients.

### Measures

The questionnaire included detailed demographics, background history and psychometric scales including the Pittsburgh Sleep Quality Index (PSQI), the Hospital Anxiety and Depression Scale (HADS) and the Perceived Stress Scale (PSS).

### Demographics

Information about the participants’ age, sex, marital status, years of experience, types of institution (hospital or primary setting) and comorbidities were obtained in the survey. In addition, participants were asked the three following questions: (1) Do you have confirmed COVID-19? (Yes/No) (2) Do any family members have suspected or confirmed COVID-19? (Yes/No) (3) Do you come into contact with confirmed or suspected COVID-19 patients in your workplace? (Yes/No).

### Sleep disturbance scale

The PSQI self-rated questionnaire assesses sleep quality over the past month [10]. The PSQI is a 19-item instrument that is categorised into seven components: subjective sleep quality, sleep latency, sleep duration, habitual sleep efficiency, sleep disturbances, use of sleep medications and daytime dysfunction. The score for each of the seven components can range from 0 to 3. The PSQI global score is calculated by summing the seven components, which a possible range from 0 to 21, with a global score of ≥ 6 indicating poor sleep quality in the previous month. The PSQI has acceptable reliability in Arabic (Cronbach’s *α* = 0.77) [11].

### Stress scale

The PSS [12], a self-administered questionnaire, was used to measure post-traumatic stress disorder (PTSD). The PSS consisted of 10 items; each item response ranged from 0 (never) to 4 (very often), with scores ranging from 0 to 40 for the total score of the scale. Scores ≥ 14 indicate the presence of stress [13]. The PSS revealed an internal consistency of 0.90 [14].

### Depression and anxiety scale

The HADS includes 14 items assessing anxiety (7 items) and depression (7 items). These are rated using a 4-point Likert-type response (from 0 to 3) [15]. The scores in each subscale are computed by summing the corresponding items, with a maximum score of 21 for each subscale. The recommended cut-off values are  ≥ 8 for both anxiety and depression [16]. The HADS showed very good internal consistency (Cronbach’s *α* = 0.83) [17].

### Data analysis

The data were entered into the Statistical Package for Social Sciences (SPSS) version 25. To address the research questions, descriptive statistics were calculated in the form of means, standard deviations, standard errors, frequencies, and percentages with regard to all the scales, subscales and participant variables. Chi-square (or Fisher’s exact test) was used to compare good and poor sleeper groups in terms of demography and stress, anxiety, and depression.

Logistic regression analyses with a full entry model were used to identify independent factors (age, sex, marital status, years of experience, type of institution (i.e., hospital or primary care), confirmed with COVID-19, family member with suspected or confirmed COVID-19, stress, anxiety, and depression) to determinants of poor sleepers (global PSQI score ≥ 6). Odds ratios (OR) and 95% confidence intervals (CI) are reported. The *p* > 0.05 was considered to be statistically significant for all analyses.

### Ethical considerations

Ethical permission was sought from Ministry of Health in Oman (MoH/CSR/20/23761). The confidentiality and privacy of the participants were maintained. A consent statement was obtained from all participants as it was presented on the first screen of the survey tool (Table 1).

## Results

A total of 987 valid questionnaires were finally received through the use of the online survey. The majority of the frontline nurses were female (90.7%, *n* = 895), and were married (85%, *n* = 839). The largest age group was those aged 31–40 years (59.5%, *n* = 587), followed by 41–50 years (20.1%, *n* = 198). Approximately two-thirds of the participants were working in a hospital setting (73.3%, *n* = 723). Around 30% of the participants had 6–10 years of experience. Only 15.1% of the participants were diagnosed with a chronic disease. Around one-quarter of the frontline nurses had confirmed or suspected COVID-19 within their family or among relatives (24.5%, *n* = 242). Seventy-one of the participants (7.2%) were confirmed to have contracted COVID-19.

**Fig. 1:**
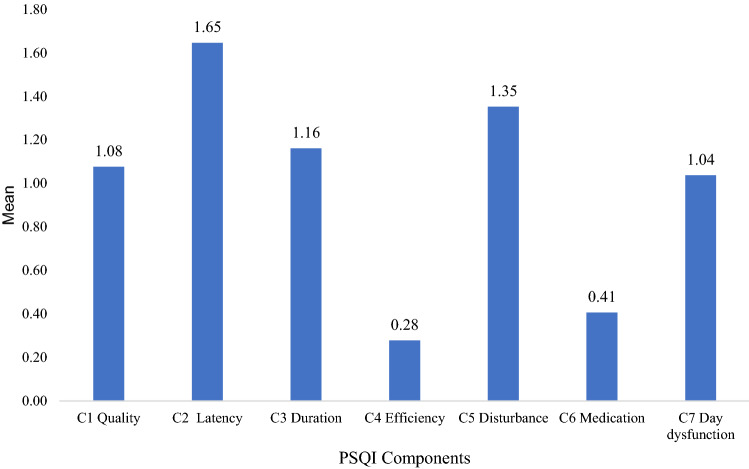
Mean of Pittsburgh Sleep Quality Index (PSQI) Components

In the total group, the mean of sleep disturbance was 6.96 (SD. 3.97, 0–21). Of the 987 participants, 580 (58.8%) experienced poor sleep quality, and 407 (41.2%) considered themselves to be good sleepers. As shown in Fig. 1, the PSQI component mean score revealed that sleep efficiency displayed the lowest score (*M* = 0.28, SD = 0.65) indicating relatively good sleep, whereas the worse component, sleep latency, displayed the highest score (*M* = 1.65, SD = 0.97).

An examination of individual PSQI and component scores revealed that the average sleep duration was 7.04 (SD = 1.59) hours per night with 61.3% (*n* = 605) of participants reporting sleep of less than 7 h. The mean of sleep latency was 38 min, with 71.4% (*n* = 705) of the participants reporting more than 15 min. Almost one-quarter (23.7%) of the participants reported a sleep efficiency of less than 85%. Thirty percent of the participants rated their quality of sleep as “fairly bad” or “very bad”. The two most common reasons for sleep disturbance was “could not get to sleep within 30 min”, and “waking up in the middle of the night or early morning” as reported by 56.03% and 54.31%, respectively (see Table 2). A considerable proportion of the frontline nurses had symptoms of stress (77.4%, *n* = 764), anxiety (47.6%, *n* = 470), and depression (42.2%, *n* = 417).

**Table 1:** Demographic and clinical characteristic of participants (*n* = 987)

	n	%	Good sleeper (*n* = 407, 41.2%)		Poor sleeper (*n* = 580, 58.8%)		*p*
			n	%	n	%	
Gender							.98
Male	92	9.3	38	9.3	54	9.3	
Female	895	90.7	369	90.7	526	90.7	
Age							.004
18–30	170	17.2	54	13.3	116	20	
31–40	587	59.5	245	60.2	342	59	
41–50	198	20.1	88	21.6	110	19	
More than 50	32	3.2	20	4.9	12	2.1	
Marital status							.001
Married	839	85.0	367	90.2	472	81.4	
Single	129	13.1	35	8.6	94	16.2	
Others	19	1.9	5	1.2	14	2.4	
Working in							.06
Hospitals	723	73.3	311	76.4	412	71	
Primary Health Care	264	26.7	96	23.6	168	29	
Years of experience							.02
< 2	99	10.0	47	11.5	52	9	
3–5	137	13.9	68	16.7	69	11.9	
6–10	296	30.0	115	28.3	181	31.2	
11–15	195	19.8	84	20.6	111	19.1	
16–20	176	17.8	56	13.8	120	20.7	
˃ 20	84	8.5	37	9.1	47	8.1	
Comorbidities							.00
Yes	149	15.1	42	10.3	107	18.4	
None	838	84.9	365	89.7	473	81.6	
Families or relatives suspected or confirmed							.00
Yes	242	24.5	76	18.7	166	28.6	
No	745	75.5	331	81.3	414	71.4	
Are you confirmed of COVID-19							.80
Yes	71	7.2	28	6.9	43	7.4	
No	916	92.8	379	93.1	537	92.6	
PSS							.00
No Stress (PSS < 14)	223	22.6	166	40.8	57	9.8	
Stress (PSS ≥ 14)	764	77.4	241	59.2	523	90.2	
HADS anxiety							.00
No anxiety (HADS(A) < 8)	517	52.4	295	72.5	222	38.3	
Anxiety (HADS(A) ≥ 8)	470	47.6	112	27.5	358	61.7	
HADS depression							.00
No depression (HADS(D) < 8)	570	57.8	301	74	269	46.4	
Depression (HADS(D) ≥ 8)	417	42.2	106	26	311	53.6

**Table 2:** Reason for sleep disturbance

Reason	≥ 1 times/Week	
	n	%
Cannot get to sleep within 30 min	553	56.03
Wake up middle or early Morning	536	54.31
Use bathroom	486	49.24
Too hot	379	38.4
Pain	346	35.06
Bad dreams	263	26.65
Too cold	169	17.12
Cannot breathe comfortably	157	15.91
Cough or snore loudly	152	15.4

There were differences in reports of poor sleep among those aged between 18 and 30 (20% vs. 13.3%), single individuals (16.2% vs. 8.6%), Others (widowed or divorced or separated) (2.4% vs. 1.2%), working in primary healthcare (29% vs. 23.6%), nurses with 6–10 (31.2% vs. 28.3%) and those with 15–20 (20.7% vs. 13.8%) years of experience, those with chronic diseases (18.4% vs. 10.3%), and those with family members with suspected or confirmed COVID-19 ( 28.6% vs. 18.7%) (*p* < 0.05) compared to good sleepers.

Poor sleep was more prevalent among nurse with stress (90.2% vs. 59.2%, *p* < 0.001), anxiety (61.7% vs. 27.5%, *p* < 0.001), and depression (53.6% vs. 26%, *p* < 0.001), when compared with the good sleeper group.

All the variables were selected to form a logistic regression model. The result was statistically significant, *χ*^2^ (19, *n* = 987) = 228.6, *p* < 0.001, which indicated that the model was able to identify effects of independent variables. The model explained between 20.7% (Cox and Snell R square) and 27.9% (Nagelkerke R square) of the variance of the independent variables. The overall correction prediction was 71%. The Hosmer–Lemeshow test supported the model (*χ*^2^ = 7.66, *p* = 0.467).

The result of the logistic regression analyses showed that none of the independent variables (gender, institution type, those with chronic diseases, family member with suspected or confirmed COVID-19, and participants with confirmed COVID-19) had significant effects on poor sleep (Table 3). Stress was the strongest factor affecting poor sleep (OR, 3.95; 95% CI: 2.73–5.72; *p* ˃0.01). Age group 18–30 appeared to be the second factor affecting poor sleep (OR, 3.52; 95% CI: 1.17–10.54; *p* = 0.02). The models showed that single marital status, less than two years and between three and five years’ experience, with anxiety and depression, were significant factors of poor sleep.

**Table 3:** Logistic regression analyses of factors associated with poor sleep quality odds ratio (95% CI)

Variables	OR	95% CI	*P*
Gender			
Male	Ref		
Female	1.08	0.66–1.78	.76
Age			
More than 50	Ref		
18–30	3.52	1.17–10.54	.02
31–40	2.47	0.92–6.63	.07
41–50	2.37	0.93–6.02	.07
Marital status			
Married	Ref		
Single	1.96	1.21–3.20	.01
Others	2.64	0.75–9.3	.13
Working In			
Primary Health Care	Ref		
Hospitals	0.97	0.69–1.3	.84
Years of experience			
˃ 20	Ref		
< 2	0.40	0.17–0.97	.04
3–5	0.45	0.21–0.98	.04
6–10	0.90	0.45–1.81	.77
11–15	0.79	0.40–1.57	.51
16–20	1.17	0.59–2.31	.65
Comorbidities			
Yes	Ref		
None	1.42	0.92–2.19	.11
Families or relatives suspected or confirmed			
No	Ref		
Yes	1.09	0.75–1.58	.66
Are you confirmed of COVID-19			
No	Ref		
Yes	1.06	0.58–1.92	.86
PSS			
No stress (PSS < 14)	Ref		
Stress (PSS ≥ 14)	3.95	2.73–5.72	.001
HADS anxiety			
No anxiety (HADS(A) < 8)	Ref		
Anxiety (HADS(A) ≥ 8)	2.27	1.62–3.17	.001
HADS depression			
No depression (HADS(D) < 8)	Ref		
Depression (HADS(D) ≥ 8)	1.48	1.05–2.07	.02

## Discussion

This study finds that sleep is frequently problematic for frontline nurses during the COVID-19 pandemic. The main finding from this study demonstrated that almost three-fifths (58.8%) of the frontline nurses had poor sleep quality, which was much higher than the 20–39% found during ordinary times [18, 19]. In addition, 77.4%, 47.6% and 42.2%, of frontline nurse had stress, anxiety, and depression.

These results were higher than a previous study from China, which reported an incidence of 38% poor sleep in the case of frontline nurses exposed to COVID-19 [20], whereas another study conducted in Italy found that only 11% of nurses had reported poor quality sleep during the COVID-19 pandemic [21]. In a recent systematic review and meta-analysis of 18 studies involving 10,697 nurses, Al Maqbali et al., [22]found the prevalence of sleep disturbance 43%, and studies included in the review reported varied prevalence of sleep disturbance between 12% and 87% during the COVID-19 pandemic.

In comparison with the general population, the current study findings were that the incidence of poor sleep was higher. For example in France it was 19.1% [23], in China 24.6% [24], and in Italy (42.2%) [25]. This difference may be partially explained by the different isolation measures that were applied by countries in their attempts to reduce the spread of COVID-19. In addition, differences in cultural norms, beliefs and values between countries may affect the prevalence of sleep disturbance. Another possible reasons for the differences are the timing of data collection and the instruments (cut-off) used. In addition, this study was conducted in August 2020 and the peak period of the COIVD-19 outbreak in Oman was July 2020, which might have increased the associated factors of sleep disturbance, stress, anxiety and depression.

In this study, 61.3% of the participants slept less than 7 h. These result were high compared to a study conducted by Ahmed et al., [26] which involved 2,095 of the general Arab population which found that 33.8% of the respondents were slept for a relatively short period (< 7 h). However, the National Sleep Foundation recommended at least 7–9 h of sleep per day for adults [27]. In fact, 60% of the participants in this study were awake before 6:30 am. Most of the nurses in our study sample were Muslim. Given that that are required to pray five times a day, the first prayer (Fajr) being one–one and a half hours before sunrise, this may the reason why most of the participants were awake before 6 am.

In addition, the results suggest that sleep latency found using the PSQI was the most disturbing component having the highest mean score compared to the other six components with an average of 38 min. Schutte-Rodin et al. [28] suggested that taking more than 30 min to fall asleep can be considered to be evidence of insomnia. This study found that the poor sleep quality of frontline nurses was attributed to such aspects as insomnia, early morning wakening, and being too hot or cold. This is consistent with previous studies which found an association between pain [29], early morning awakening [30], being too hot or cold [31] and disturbance of sleep among nurses.

The present study indicates that frontline nurses with at least one chronic disease had the highest levels of sleep disturbance. This may be because comorbidities are highly prevalent among fatal cases of COVID-19 [32, 33]. The present study demonstrates that age between 18 and 30, being single, and having less than 5 years’ experience were predicators of poor sleep. Several researchers have suggested that a lack of skills, knowledge and experience may have an influence on the increased prevalence of sleep disturbance among nurses under 35 years of age [34–36].

In the current study, a significant association was found between poor sleep and stress, anxiety, and depression. Likewise, the risk of being stressed, anxious, and depressed significantly predicted the poor sleep quality. A similar result was reported by Lai et al., [20], Yin et al., [37] and Shechter et al. [38], among nurses during the COVID-19 pandemic. Understanding the underlying causes of sleep disturbance is essential with regard to introducing interventions that can help to reduce the symptoms of frontline nurses during a pandemic.

The study has a number of limitations. First, the study utilized a cross-sectional design; therefore, it represents the evaluation of stress, anxiety, depression and poor sleep disturbance at one point in time, without any longitudinal observation of the participants. Second, the participants were recruited from Oman, which may limit generalization to other countries. Finally, the study relied on the use of self-reporting questionnaires to assess the extent of sleep disturbance; however, this may differ from an objective sleep assessment [39].

## Conclusion

Sleep disturbance is a significant problem for frontline nurses treating patient with COVID-19 in Oman. Sleep disturbance is significantly related to increases in stress, anxiety, and depression in terms of the participating group. This study helps to improve understanding of the sleep disturbance of frontline nurses exposed to an outbreak of a fast-spreading, life-threatening infectious disease, and could help strengthen preparations for responding to possible future outbreaks or infectious disease pandemics. Furthermore, it provides a solid foundation for the next step in the research, which aims to identify appropriate interventions to improve the sleep quality and psychological wellbeing of frontline nurses.
